# The Relationship Between Cortical Inhibition and Electroconvulsive Therapy in the Treatment of Major Depressive Disorder

**DOI:** 10.1038/srep37461

**Published:** 2016-12-09

**Authors:** Daphne Voineskos, Andrea J. Levinson, Yinming Sun, Mera S. Barr, Faranak Farzan, Tarek K. Rajji, Paul B. Fitzgerald, Daniel M. Blumberger, Zafiris J. Daskalakis

**Affiliations:** 1 Centre for Addiction and Mental Health, Toronto, Ontario, Canada; 2 Department of Psychiatry, University of Toronto, Toronto, Ontario, Canada; 3 Health & Wellness Centre, The University of Toronto, Ontario, Canada; 4 Department of Electrical and Computer Engineering, University of Toronto, Toronto, Ontario, Canada; 5 Monash Alfred Psychiatry Research Centre, Alfred and Monash University Central Clinical School, Victoria, Australia

## Abstract

Dysfunctional cortical inhibition (CI) is postulated as a key neurophysiological mechanism in major depressive disorder. Electroconvulsive therapy (ECT) is the treatment of choice for resistant depression and ECT has been associated with enhanced CI. The objective of this study was to evaluate the relationship between CI and ECT response in resistant depression. Twenty-five patients with treatment resistant depression underwent an acute course of ECT. CI was indexed by the cortical silent period (CSP) and short-interval cortical inhibition (SICI), through TMS-EMG. CI and clinical response was measured prior to beginning an acute ECT course and within 48 hours of the last ECT treatment in the course. Clinical response to ECT was assessed by HDRS-17 before and after an acute course of ECT. We found that there was a significant difference in CSP at baseline between responder and non-responder groups (p = 0.044). Baseline CSP predicted therapeutic response to ECT with sensitivity of 80% and specificity of 60%. There were no changes in CSP or SICI after administration of the ECT course. Our findings suggest that duration of pre-treatment CSP may be a useful predictor of therapeutic response to ECT in patients with TRD.

Major Depressive Disorder (MDD) is highly prevalent, impacting 6.7% of Americans annually^1^. Unfortunately, current first line treatments, including antidepressant medications, fail to achieve remission in 1 out of 3 patients with MDD^2^. Once two adequate antidepressant trials have been unsuccessful, the illness is termed treatment resistant depression (TRD)^3^. Electroconvulsive therapy (ECT) is the most effective treatment for patients with TRD^4^.

A course of ECT for an acute episode of depression generally occurs two or three times per week for up to 15–18 treatments. During each treatment, a series of high frequency electrical pulses are delivered to either the non-dominant right hemisphere and vertex (i.e., unilateral ECT) or bilaterally (i.e., bitemporal or bifrontal ECT). In ECT, repetitive electrical stimulation over the cortex results in an entrainment of pyramidal cell firing with subsequent generalization of cortical activity. This produces a generalized tonic-clonic seizure, which typically self-terminates within 30–60 seconds. A report from the Consortium for Research in ECT (CORE)^5^ revealed that over half of the subjects with a depressive illness who were treated with ECT had improved clinically within one week, and after ten treatments, 65% had achieved symptom remission. Other studies have reported that over 50% of patients who have failed to respond to one or more adequate antidepressant medication trials respond to ECT^6^. Meta-analyses reinforce the superiority of ECT in the treatment of depressive episodes over sham ECT, placebo or antidepressant medications^4^,^7^. While ECT has profound neurophysiological effects, owing to its ability to produce seizures, the precise biological mechanisms underlying the neurophysiological effects have yet to be elucidated^8^,^9^.

One postulated mechanism through which ECT may exact its therapeutic effect is through cortical inhibition (CI). CI is defined as the neurophysiological process in which γ-aminobutyric acid (GABA) inhibitory interneurons modulate cortical neuronal activity, through connections to pyramidal neurons as well as other interneurons. Numerous investigations have suggested that MDD symptoms are closely associated with deficits in GABAergic inhibitory neurotransmission. As such, aberrant CI in MDD has been demonstrated through several investigational techniques. For example, a neuropathologic study by Rajkowska *et al*.^10^, which examined post-mortem sections from the dorsolateral prefrontal cortex (DLPFC) and orbitofrontal cortex in 14 subjects with MDD and 11 controls, demonstrated reduced density of cortical GABA interneurons in the prefrontal cortex of patients with MDD. Results from neuroimaging studies also demonstrated reductions in cortical levels of GABA in MDD. Sanacora *et al*.^11^ used *in-vivo* imaging techniques (specifically proton magnetic resonance spectroscopy) to measure GABA levels in the occipital cortex of 14 medication-free MDD subjects. They found that the depressed group had a significant reduction (52%) of GABA compared to healthy subjects. The same group was able to demonstrate that treating MDD patients with ECT or antidepressant medications significantly improved low levels of GABA in the occipital cortex^12^,^13^.

Transcranial Magnetic Stimulation (TMS) is a neurophysiological investigative tool utilizing electromagnetic induction to induce currents in brain tissue. TMS provides an index of GABA receptor-mediated inhibition in the cortex as it differentially stimulates inhibitory interneurons and pyramidal neurons. There are several TMS paradigms that provide a measure of GABA receptor-mediated inhibitory neurotransmission, however, this study will focus specifically on the cortical silent period (CSP) and short interval cortical inhibition (SICI). The CSP is measured by stimulating the motor cortex on superimposed background electromyography (EMG) activity. At high stimulus intensities, a cessation of all EMG activity occurs (thus the ‘silent’ period). It is the duration of this ‘silent’ period, measured until the return of EMG activity, which provides a metric of GABAergic inhibition in the cortex^14^. CSP appears to be mediated by cortical GABA_B_ interneurons, as evidenced by several investigations^15^,^16^,^17^,^18^. For example, subjects who were administered baclofen, a GABA_B_ agonist, demonstrated increased CSP duration^19^, suggesting that this neurophysiological mechanism is coordinated through GABA_B_ receptor mediated inhibitory neurotransmission. SICI, in contrast, includes a subthreshold conditioning pulse followed by a suprathreshold test pulse. This TMS paradigm has been shown to index GABA_A_ receptor mediated inhibition^14^,^20^. For example, SICI is enhanced by benzodiazepines, which facilitate GABA_A_ receptor mediated inhibitory neurotransmission^14^. Previous studies have reported deficits in CSP and SICI in MDD^21^.

TMS measures of CI have been evaluated in response to ECT^22^. Following a report on 2 patients^23^, Bajbouj *et al*.^22^ compared CI measures in 10 patients before and after 10 sessions of right unilateral ECT, and found significant increases in CI measures after the course of treatment. Specifically, both SICI and CSP duration were increased after the final ECT session as compared to baseline measures, despite no significant changes in resting motor threshold (RMT) or intracortical facilitation (ICF) measures^22^. This dovetails with one of the predominant theories; that ECT exerts its antidepressant effects primarily by enhancing the GABAergic activity of the brain, as demonstrated by the anticonvulsant effects of ECT.

Collectively, the studies described above suggest that potentiation of CI, as evaluated by CSP and SICI measures, may represent a unique neurophysiological mechanism through which ECT exerts its therapeutic effects in TRD. To date, however, no studies have evaluated a direct link between CI and response to an acute course of ECT. We hypothesized that CI may predict treatment response to ECT in patients with TRD. We also hypothesized that we will replicate previous findings from other studies^22^ demonstrating prolongation of the CSP in response to an acute course of ECT.

## Results

### Demographics

The sample consisted of nineteen women and six men with a mean age of 47.4 ± 15.25 years. There were fifteen responders and ten non-responders to ECT, with response defined as a decrease in HDRS-17 score after ECT treatment by ≥50%. The mean change in HDRS-17 score overall was 9.92 ± 8.34 and was 15.60 ± 4.75 for responders and 1.40 ± 3.98 for non-responders. Within the responder group, the mean age was 42.93 years (range of 19–67, ± 16.59 years) with 3 men and 12 women. Within the non-responder group, the mean age was 53.20 years (range of 29–72, ± 12.02 years) with 3 men and 7 women. An independent t-test determined there was no significant difference in age between the responder and non-responder group (t(23) = 1.680, p = 0.106). A further independent t-test was performed to assess potential differences in HDRS-17 scores at baseline between responder (25.27 ± 4.49) and non-responder (22.80 ± 2.04) groups, but no significant differences were determined (t(24) = −1.857, p = 0.078). All 25 subjects began treatment with right unilateral ultrabrief ECT, and 9 subjects switched to bitemporal ECT partway through their courses of treatment. This switch occurred if the subjects showed poor initial response to treatment. Demographics are displayed in Table 1, neurophysiological measurements are detailed in Table 2.

### Cortical Silent Period

**Figure 1:**
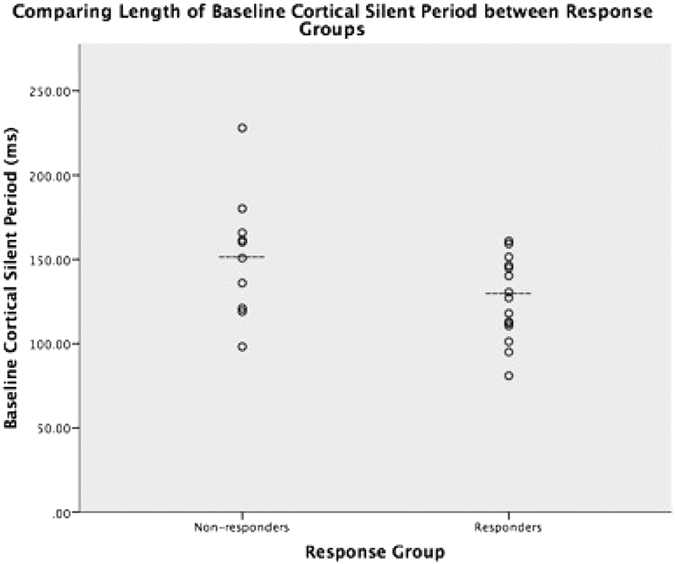
Comparing Baseline CSP between Response Groups. Dashed line (---) denotes mean for each group.

**Figure 2:**
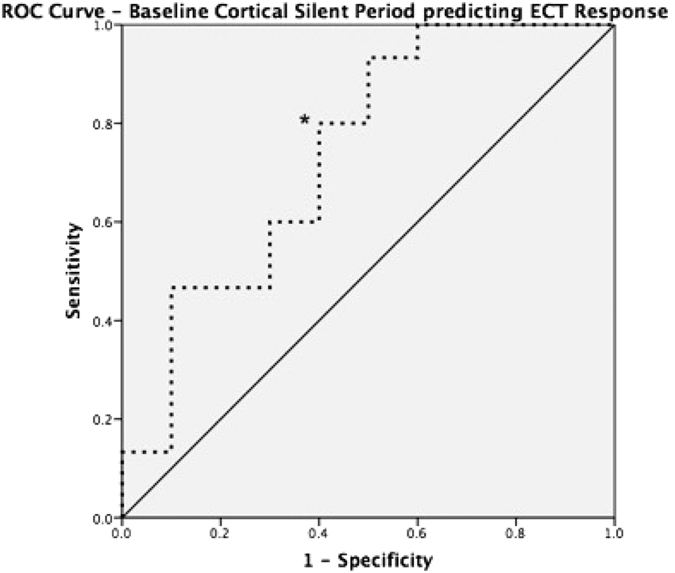
Receiver Operating Characteristic plotting Baseline CSP prediction of ECT response. *Denotes maximal 80% Sensitivity, 60% Specificity (AUC = 0.74, p = 0.046).

The mean CSP (in milliseconds (ms)) for the entire sample did not show significant change from baseline (136.50 ± 31.88) to the post ECT treatment course (141.98 ± 37.44), (t(24) = −0.663, p = 0.514). There was no significant correlation between change in CSP and percentage change in HDRS-17 (Pearson’s r = 0.385, p = 0.057). Baseline CSP was found to be normally distributed across the sample (W(25) = 0.956, p = 0.342). The mean CSP for the responder group was 126.12 ± 24.26 ms at baseline (Fig. 1) and 142.33 ± 39.39 ms after ECT treatment course. A paired samples t-test did not show significant change in CSP length with ECT treatment (t(14) = −1.465, p = 0.165) in the responder group. The mean CSP for the non-responder group was 152.07 (±36.70) ms at baseline (Fig. 1) and 141.46 (±36.40) ms after ECT treatment course. A paired samples t-test also did not show significant change in CSP length with ECT treatment in the non-responder group (t(9) = 0.959, p = 0.363). A repeated measures ANOVA was performed and showed no significant interaction between response and CSP length (F = 1.724, p = 0.222), which was likely due to there being no significant CSP lengthening with ECT treatment. An independent t-test demonstrated a difference between the CSP measurement at baseline between the two response groups (t(23) = 2.136, p = 0.044) (Fig. 1). A power calculation was performed for this analysis, utilizing the mean duration of CSP of each response group at baseline, with the overall sample size at 25 and overall standard deviation σ = 31.88, type I error rate, α = 5%, and sampling rate, κ = 1.5 (given responder group n = 15 and non-responder group n = 10). With these values, the power, defined as 1-β, was found to be 0.91. There was no correlation between baseline CSP length and any investigated clinical variables within each response group. A receiver operating characteristic (ROC) analysis was performed to assess the value of baseline CSP in predicting response to ECT. Across the entire sample, a sensitivity of 80% and specificity of 60% (AUC = 0.74, p = 0.046) was observed (Fig. 2).

### CSP and Length of Seizure

Seizures were monitored visually with a timer and on 2-lead EEG until termination was noted both visually and on EEG. Seizure duration is defined as length of motor/peripheral seizure activity at the first treatment after determination of the seizure threshold. The mean seizure duration for the entire sample was 40.67 ± 15.20 seconds. The mean duration of seizure in non-responders was 35.60 ± 13.55 seconds and in responders was 44.29 ± 15.73 seconds. There was no difference between the 2 groups in terms of mean duration of seizure (t(22) = −0.1410, p = 0.172). A Pearson’s analysis did not show significant correlation between baseline CSP and length of seizure (Pearson’s r = 0.329 p = 0.116).

### Short Interval Cortical Inhibition and Intracortical Facilitation

The mean SICI for the entire sample did not show significant change from baseline (70.37 ± 37.19%) to post ECT treatment course (80.20 ± 45.52%), (t(24) = −1.00, p = 0.33). There was no significant difference in SICI at baseline between responder (64.80 ± 39.91%) and non-responder (78.71 ± 32.88%) groups (t(24) = 0.913, p = 0.371). There was no significant difference in SICI post-ECT treatment course between responder (76.50 ± 48.02%) and non-responder (85.74 ± 43.47%) groups (t(23) = 0.489, p = 0.629). Further, there was no significant correlation between change in SICI and percentage change in HDRS-17 (Pearson’s r = −0.005, p = 0.982). The mean ICF at baseline in the entire sample was 159.70 (±100.11)%, and mean ICF post-ECT treatment was 170.67 (±90.45)%. Again, there was no significant change in ICF with ECT treatment (t(24) = −0.495, p = 0.625).

## Discussion

This study produced a few key findings. First, in patients with TRD, we found significant differences between baseline CSP length in ECT responders versus ECT non-responders (Fig. 1). Further examination of this data through a ROC analysis demonstrated that baseline CSP predicted ECT response with a sensitivity of 80% and specificity of 60% (Fig. 2). Finally, and importantly, there was no significant association between baseline CSP length with seizure duration or a change in CSP duration with ECT treatment, in contrast to previous studies.

Several studies have examined cortical excitability and inhibition in relation to ECT in TRD. Increased cortical excitability has been described in a case report of one subject^24^ and after 6 bitemporal ECT treatments in combination with active or sham repetitive TMS (rTMS)^25^. However, these studies did not assess changes in the CSP. Rather, they focused on the active motor threshold and SICI/ICF. More recently, a study of 10 right unilateral ECT sessions in 10 right-handed patients with MDD found an increase in CSP and increased SICI after the treatment course^22^. An important negative finding of our study there was no significant change in the CSP duration or SICI following an acute course of ECT. There may be several reasons for this. First, our considerably larger sample size of 25 patients, compared to 10 patients in the Bajbouj *et al*. study, may have contributed to an attenuated neurophysiological effect. Second, previous studies have measured CI after a set number of ECT treatments (i.e. 6–10 treatments), while our study titrated treatment number to response. Greater individualized number of ECT treatments may demonstrate a different overall effect on CI. Moreover, previous studies have examined the effects of either right unilateral or bitemporal ECT treatments, whereas patients in this study may have had either right unilateral ultrabrief ECT, bitemporal standard pulse ECT or both, sequentially, to enhance the likelihood of individual response. Importantly, the Bajbouj *et al*.^22^ study utilized brief pulse right unilateral ECT, usually delivered with a pulse width of 0.5–1.0 ms, whereas all subjects in our study began treatment with right unilateral ultrabrief ECT, which is delivered in our clinic with a pulse width of 0.3 ms, closer to physiological neuronal depolarization. This has been shown to avoid excessive stimulation during neuronal refractory periods^26^. As non-responders were more likely to switch to bitemporal standard pulse ECT in a bid to increase efficacy of the treatment, this may have been a confounding factor in our study. We postulate that the effects found here may be related to different types of ECT treatment. Finally, the mean age of our sample was approximately 7 years younger than that of Bajbouj *et al*.^22^, which may be a contributing factor, as cortical inhibitory measures are known to change with increasing age^27^,^28^.

We demonstrated that a shorter baseline CSP was a significant predictor of symptom response. That is, the CSP predicted treatment response to an acute course of ECT with an 80% sensitivity and 60% specificity. The ability of the CSP to predict ECT treatment response highlights the role of interneuronal networks involved in the clinical response to ECT. This is consistent with the findings that lower GABA concentrations in the cortex may result in greater seizure propensity and a greater likelihood of an ECT induced antidepressant response. As GABA has inherent anticonvulsant properties^29^, it follows that reduced interneuronal GABA may facilitate ECT’s ability to produce large-scale synchronization of pyramidal cell activation (i.e., a seizure) with multiple repeated stimuli. In support of such findings, benzodiazepines have been found to potentiate both GABA^30^ and the CSP^31^. Furthermore, concurrent benzodiazepine use has been associated with poor ECT outcomes. An interesting aspect to our findings is the disparity between the findings in CSP and SICI. The CSP has been linked to GABA_B_ receptor mediated inhibitory neurotransmission and SICI to GABA_A_ receptor mediated inhibitory neurotransmission. Both GABA_A_ and GABA_B_ receptors have been implicated in depression^10^,^11^, however the two receptor subtypes have been shown to have different neurophysiological effects: GABA_A_ receptors act as ligand-gated ion channels^32^,^33^, while GABA_B_ receptors are metabotropic, long acting and modulate both LTP and excitatory postsynaptic potentials^34^. Previous studies reported that ECT has specific effects on GABA_B_ receptor mediated inhibition as measured through blood serum levels and baclofen challenge^35^. As such, we postulated that ECT would result in GABA_B_ receptor modulation as indexed through TMS. However, further studies may wish to evaluate the effects of ECT on LTP via TMS-PAS^36^ to better understand the neurophysiological effects of ECT in the cortex. Collectively, these results suggest that CSP may be used to predict response to ECT and highlight the potential role of GABA_B_ receptor mediated inhibitory neurotransmission as a potential biomarker of response to seizure therapies in depression. Whether such findings are demonstrated in larger ECT treatment samples or with non-convulsive modalities of brain stimulation therapy (ie. rTMS, transcranial direct current stimulation (tDCS)) remains to be determined.

If replicated, these results may be used to guide ECT treatment and also to generate a better understanding of the neurobiology and treatment of TRD. For example, the CSP may be used to preselect which patients are most likely to benefit from ECT based on the high sensitivity of our findings. Additionally, there is some recent evidence suggesting that anodal tDCS may reduce the CSP^37^. This raises the possibility that tDCS may be used pretreatment to shorten the CSP and, in turn, potentially enhance the efficacy of ECT.

A limitation of our study may be the relatively small sample size and lack of replication of the results of other investigations into CI and ECT response. Furthermore, in our study, CI was measured within 48 h of the final ECT treatment as opposed to within 24 h of the final ECT treatment, as in Bajbouj^22^. There may be scientific value in serial CI measurements after ECT (spanning from the day of treatment to several days after) to examine the downstream inhibitory and excitatory neurophysiological effects of ECT. Additionally, future studies may wish to probe CI measures in cortical regions most closely associated with the pathophysiology of depression (i.e., the DLPFC) by measuring the neurophysiological equivalent of the CSP (i.e., N100) from the DLPFC^38^. Nevertheless, the impact of this work has the potential to inform our understanding of TRD illness and more importantly, there is a potential to guide the decision to pursue an invasive treatment like ECT, based on a simple neurophysiological test. Overall, further understanding of the neurophysiology of treatment resistance and brain stimulation therapies may lead to more personalized and efficient care.

## Methods

### Subjects

Twenty-five patients with a DSM-IV diagnosis of MDD referred for ECT treatment to the Brain Stimulation Treatment and Research Program at the Centre for Addiction and Mental Health were recruited. Diagnosis was confirmed by the Structured Clinical Interview for DSM-IV (SCID)^39^. Patients were excluded if they: (1) had a history of DSM-IV substance dependence in the last 6 months, and had DSM-IV substance abuse in the last month, (2) had a concomitant major unstable medical or neurologic illness or have had a history of seizures, (3) were pregnant, (5) had metal implants, (6) had a co-morbid borderline personality disorder and/or antisocial personality disorder as confirmed by the SCID for Axis II Disorders (SCID-II)^39^, which may prevent the patient from completing the procedures required for the study; (7) had a positive urine toxicology screen for drugs of abuse. All 25 patients had a history of medicated TRD, defined as having failed to achieve a meaningful clinical response (ie. Hamilton Depression Rating Scale (HDRS-17) >22) after at least 2 separate antidepressant trials of sufficient dose for at least 6 weeks according to stage II criteria outlined by Thase and Rush^40^. Demographic and clinical data are listed in Table 1. The Centre for Addiction and Mental Health Ethics committee approved the study in accordance with the declaration of Helsinki and written informed consent was obtained for each participant. The study methods were carried out in accordance with the Ethics Committee regulations of the Centre for Addiction and Mental Health.

### ECT Treatments

ECT was administered with a square-wave, constant-current, brief-pulse device (MECTA Corporation, Lake Oswego, OR). ECT was administered open label two or three times per week according to our centre’s protocol. The seizure threshold was determined at the first treatment using the previously published titration procedure^41^. For all subsequent treatments, stimulus intensity was delivered at either 1.5 times the seizure threshold (in bitemporal ECT) or 6 times the seizure threshold (in right unilateral ultra brief ECT). The decision to administer right unilateral ultra brief ECT or bitemporal ECT was made based on treating physician/patient preference, and the electrodes were placed according to guidelines outlined by the American Psychiatric Association^42^. Methohexital and succinylcholine were the typical anaesthetic medications used, with dosing guidelines of 0.75–1.0 mg/kg methohexital and 0.4–0.6 mg/kg succinylcholine. Treatment termination was based on response, clinical factors and/or the patient’s expressed wish to discontinue ECT.

### Clinical Measures

Demographic variables and potential covariates were recorded at baseline following a clinical interview, including the severity of the current episode, comorbid psychiatric and physical diagnoses, classes of current medications, seizure length of the first treatment after threshold achieved and type and dose of current and previous treatment. Clinical measures were performed at baseline and at the end of the course of ECT treatment. The primary clinical response outcome variable was the HDRS-17. Response defined as a decrease in HDRS-17 score after ECT treatment course by ≥50%.

### Procedures

Measurement of CI (i.e., CSP and SICI) was conducted within one week prior to the initiation of the first ECT treatment and the post-ECT evaluation of CI was conducted within 48 hours after the last ECT session. There is evidence that general anaesthetic may alter neurophysiological indices in the brain^43^,^44^ in some cases up to 24 h, after ECT treatment, which could confound the results measured in this investigation. Moreover, we allowed subjects up to 48 h to return for the last measurement of neurophysiological indices to allow for variability in patient availability regarding returning to the centre for final investigations, in order to ensure as many subjects as possible completed the study. Surface electromyography (EMG) was recorded from the right abductor pollicis brevis (APB) muscle. The participant maintained relaxation throughout the experiment. TMS pulses were applied to the hand area of the left motor cortex with a figure of eight magnetic coil and Magstim 200 magnetic stimulators (Magstim, Whitland, Dyfed, Wales). The coil diameter was 70 mm for each loop.

The coil was held tangentially on the head with the handle pointing backward and 45 degrees laterally from the midline. The RMT was defined as the intensity that produced a motor evoked potential (MEP) of >50 μV in 5 of 10 trials in a relaxed APB muscle^16^. Measurement of CSP duration was obtained in moderately tonically active APB (ie. 20% of maximum contraction) by stimulating the motor cortex with intensities of 140% of RMT. Ten trials were performed at this intensity. The CSP duration was defined as the absolute CSP, i.e., the time from the MEP onset to the return of any voluntary EMG activity. The absolute CSP ends with a deflection in the EMG waveform^45^. In SICI, a subthreshold conditioning stimulus (CS), set at 80% of RMT, preceded a suprathreshold test stimulus (TS), that was adjusted to produce an average MEP of 0.5–1.5 mV peak to peak amplitude in the contralateral APB muscle^16^. Conditioning stimuli were applied to the motor cortex before the TS at one of five random ISIs: 2 msec, 4 msec for SICI; and 10 msec, 15 msec, and 20 msec for ICF. For SICI, changes in the TS MEP amplitude at each interstimulus interval (ISI) were expressed as a percentage of the mean unconditioned MEP amplitude^46^. The order of administration of the two paradigms was counterbalanced between subjects to prevent order effects.

### Statistical Analyses

Demographics, clinical symptom severity, seizure characteristics and TMS measures were compared between responders and non-responders by independent t-test (for scalar variables) and chi-squared tests (for categorical variables). As there were equal numbers of participants taking concomitant benzodiazepine medications in each response group (see Table 1), extensive statistical analyses of the effect of this medication class were not performed. Baseline RMT values were compared to post-ECT course RMT with a paired t-test. Changes in CI measures (both SICI and CSP) and ICF from baseline to after the course of ECT were examined by paired t-tests in the sample as a whole, as well as in responder and non-responder groups separately. Independent t-tests were performed to assess potential differences in CSP and SICI measures between responder and non-responder groups at the two time points (i.e. baseline and post-ECT course). The relationship of CI measures (specifically baseline CSP and post-ECT CSP) with HDRS-17 scores before and after the course of ECT were compared by Spearman’s correlation and multiple regression for the sample as a whole, as well as separately for responders and non-responders. The relationship of CSP measures with seizure characteristics were compared by Pearson’s correlation. Normal distribution of data was assessed with Shapiro-Wilk tests. All statistical procedures were two-tailed and significance was set at an alpha level of 0.05. Analyses were performed with SPSS 22 (IBM SPSS).

## Additional Information

**How to cite this article**: Voineskos, D. *et al*. The Relationship Between Cortical Inhibition and Electroconvulsive Therapy in the Treatment of Major Depressive Disorder. *Sci. Rep.* **6**, 37461; doi: 10.1038/srep37461 (2016).

**Publisher's note:** Springer Nature remains neutral with regard to jurisdictional claims in published maps and institutional affiliations.

## Figures and Tables

**Table 1:** Demographics and Results.

	Total Sample	Responders	Non-responders	*Comparing Response Groups*	
				t	(p)
*Demographics*					
**(n.)**	25	15	10		
**Sex**					
Male	6	3	3		
Female	19	12	7		
**Age (range in years)**	47.4 (19–72)	42.93 (29–68)	53.20 (29–72)	1.68	0.106
*Clinical Information*					
**HDRS-17**					
Baseline (±sd)	24.28 (±3.86)	25.27 (±4.50)	22.80 (±2.04)	−1.857	0.078
Post-treatment (±sd)	14.36 (±6.65)	9.67 (±2.77)	21.40 (±3.78)	8.977	<0.0001
Change (±sd)	9.92 (±8.34)	15.60 (±4.75)	1.40 (±3.98)	−7.795	<0.0001
**Age at Onset (range in years)**	29.83 (11–62)	26.29 (13–62)	35.33 (11–60)		
**Recurrence**					
Single Episode	1	0	1		
Recurrent	24	15	9		
**Severity**					
mild	0	0	0		
moderate	11	6	5		
severe w/o psychotic f.	10	7	3		
unknown	4	2	2		
**Medications**					
antidepressant	22	12	10		
mood stabilizer	0	0	0		
benzodiazepine	8	4	4		
antipsychotic	8	6	2		
stimulant	3	1	2		
no psychotropic medication	2	2	0		
**Psychiatric Comorbidities**					
Dysthymic Disorder	2	0	2		
Axis II Diagnosis	1	1	0		
Anxiety Disorders	9	6	3		
Other	1	1	0		
None	13	8	5		
**Physical Comorbidities**					
Endocrine	6	4	2		
Musculoskeletal/Pain	4	3	1		
Cardiovascular	2	0	2		
Other	6	4	2		
None	10	7	3		
*ECT Information*					
**Number of treatments (range)**	13.60 (8–29)	12.13 (8–21)	15.80 (9–29)		
**Seizure Threshold (mC) (±sd)**	26.18 (±10.76)	22.40 (±10.30)	30.72 (±9.91)		
**Seizure Duration** (**seconds**)					
Observed physical (±sd)	40.67 (±15.19)	44.29 (±15.73)	35.60 (±13.55)	—	—
Observed EEG (±sd)	54.87 (±21.44)	56.21 (±19.18)	53.10 (±25.26)	—	—
*Neurophysiology*					
**RMT** (**%**)					
Baseline (±sd)	48.25 (±10.36)	—	—	—	—
Post-treatment (±sd)	45.84 (±7.79)	—	—	—	—
Change (±sd)	−3.00 (±8.20)	—	—	—	—
**Cortical Silent Period** (**ms**)					
Baseline (±sd)	136.50 (±31.88)	126.12 (±24.26)	152.07 (±36.70)	2.136	0.044
Post-treatment (±sd)	141.98 (±37.44)	142.33 (±39.39)	141.46 (±36.40)	−0.055	0.956
Change (±sd)	5.48 (±41.33)	16.20 (±42.83)	−10.60 (±34.96)	−1.644	0.114
**SICI** (**%**)					
Baseline (±sd)	70.37 (±37.19)	64.80 (±39.91)	78.71 (±32.88)	0.913	0.371
Post-treatment (±sd)	80.20 (±45.52)	76.50 (±48.02)	85.74 (±43.37)	0.489	0.629
Change (±sd)	9.83 (±49.09)	11.70 (±44.04)	7.03 (±58.26)	−0.228	0.821

Values of t-tests and significance (p) denote comparisons between response groups. Only the most common psychiatric and physical comorbidities in the study population are noted in this table.

**Table 2:** Neurophysiological Measures at Baseline and Post-Treatment.

	Baseline	Post-treatment	(p)
Resting Motor Threshold (%)	48.25 (±10.36)	45.84 (±7.79)	0.14
Cortical Silent Period (ms)	136.50 (±31.88)	141.98 (±37.44)	0.51
Short Intracortical Inhibition (%)	70.37 (±37.19)	80.20 (±45.52)	0.33
Intracortical Facilitation (%)	159.70 (±100.11)	170.67 (±90.45)	0.63

Neurophysiological measures are noted for the sample as a whole.
